# Epitaxy of 2D Materials toward Single Crystals

**DOI:** 10.1002/advs.202105201

**Published:** 2022-01-17

**Authors:** Zhihong Zhang, Xiaonan Yang, Kaihui Liu, Rongming Wang

**Affiliations:** ^1^ Beijing Advanced Innovation Center for Materials Genome Engineering Beijing Key Laboratory for Magneto‐Photoelectrical Composite and Interface Science Institute for Multidisciplinary Innovation School of Mathematics and Physics University of Science and Technology Beijing Beijing 100083 China; ^2^ State Key Laboratory for Mesoscopic Physics and Frontiers Science Center for Nano‐optoelectronics School of Physics Peking University Beijing 100871 China; ^3^ Interdisciplinary Institute of Light‐Element Quantum Materials and Research Centre for Light‐Element Advanced Materials Peking University Beijing 100871 China

**Keywords:** 2D materials, epitaxy, interaction, lattice symmetry, single crystal

## Abstract

Two‐dimensional (2D) materials exhibit unique electronic, optical, magnetic, mechanical, and thermal properties due to their special crystal structure and thus have promising potential in many fields, such as in electronics and optoelectronics. To realize their real applications, especially in integrated devices, the growth of large‐size single crystal is a prerequisite. Up to now, the most feasible way to achieve 2D single crystal growth is the epitaxy: growth of 2D materials of one or more specific orientations with single‐crystal substrate. Only when the 2D domains have the same orientation, they can stitch together seamlessly and single‐crystal 2D films can be obtained. In this view, four different epitaxy modes of 2D materials on various substrates are presented, including van der Waals epitaxy, edge epitaxy, step‐guided epitaxy, and in‐plane epitaxy focusing on the growth of graphene, hexagonal boron nitride (h‐BN), and transition metal dichalcogenide (TMDC). The lattice symmetry relation and the interaction between 2D materials and the substrate are the key factors determining the epitaxy behaviors and thus are systematically discussed. Finally, the opportunities and challenges about the epitaxy of 2D single crystals in the future are summarized.

## Graphical abstract

The preparation of single‐crystal two‐dimensional materials is of great significance for their practical applications. Here, four epitaxy modes of graphene, hexagonal boron nitride and transition metal dichalcogenide on various substrates, are summarized and discussed systematically, which may provide a deeper insight into the epitaxial growth and potential opportunities to realize large‐scale production of 2D single crystals.

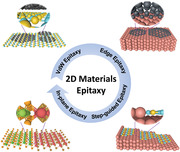

## 1 Introduction

Single crystal manufacturing has promoted the rapid development of the semiconductor industry; the large‐size high‐quality silicon (Si) ingot makes the large‐area intergraded circuit with high density and reliability possible. However, the scaling of Si based transistors has approached its physical limit as the continuous upgrading. Thus, exploiting alternative materials is currently the key to realize the smaller, faster and cheaper chips in the future. Two‐dimensional (2D) materials with thickness ranging from single‐ to few‐atomic thick layer have become the most promising candidate to replace conventional materials to construct new‐generation electronic and optoelectronic devices.^[^ ^1^, ^2^, ^3^, ^4^, ^5^, ^6^, ^7^, ^8^, ^9^, ^10^, ^11^ ^]^ A variety of device prototypes have been devised and demonstrated excellent performance and remarkable application potential.^[^ ^12^, ^13^, ^14^, ^15^, ^16^, ^17^, ^18^, ^19^, ^20^, ^21^, ^22^, ^23^ ^]^ However, the prerequisite of industrial applications of 2D materials is the fabrication of large‐area high‐quality single crystals, which would possess superior intrinsic properties and high homogeneity to meet the demands of high‐performance devices integration. Therefore, the preparation of single‐crystal 2D materials is of great significance for their practical applications.

Two strategies have been developed to grow 2D single crystals. One is to make one nucleus grow up. In this growth regium, the precursors need to be well controlled to reduce the nucleation density^[^ ^24^, ^25^, ^26^, ^27^, ^28^, ^29^, ^30^, ^31^, ^32^, ^33^, ^34^, ^35^ ^]^ or fed locally to allow only one nucleus to form and grow up,^[^ ^36^, ^37^ ^]^ always leading to slower growth rate and more energy and time consumption. Although various techniques have been developed to enhance the growth rate effectively,^[^ ^38^, ^39^, ^40^, ^41^, ^42^ ^]^ this strategy is still not suitable for the large‐scale production. The other is to make all the nuclei have the same orientation, then grow up and finally merge into a continuous film without grain boundaries.^[^ ^43^, ^44^ ^]^ Mostly it is realized by the epitaxial growth on single‐crystal substrate where millions of nuclei expand their size simultaneously and thus a large film could be obtained in a short time. Accordingly, epitaxial growth has been universally recognized as the most promising technique to realize the large‐scale preparation of single‐crystal 2D materials.

**Figure 1:**
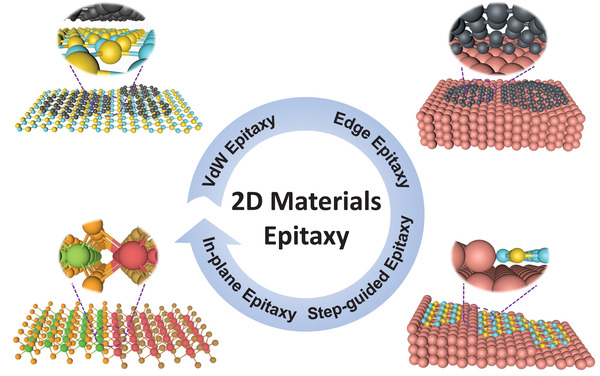
Schematic illustration of four types of epitaxial growth mode for 2D materials.

Up to now, different 2D materials have been epitaxially grown on various single‐crystal substrates, including graphene and hexagonal boron nitride (h‐BN) on copper (Cu),^[^ ^43^, ^44^, ^45^, ^46^, ^47^, ^48^, ^49^, ^50^, ^51^, ^52^ ^]^ germanium (Ge),^[^ ^53^, ^54^, ^55^ ^]^ other transitional metals^[^ ^56^, ^57^, ^58^, ^59^, ^60^, ^61^ ^]^ and metal alloys,^[^ ^62^, ^63^, ^64^, ^65^ ^]^ and transition metal dichalcogenides (TMDCs) on sapphire,^[^ ^66^, ^67^, ^68^, ^69^, ^70^, ^71^, ^72^, ^73^, ^74^ ^]^ mica,^[^ ^75^, ^76^ ^]^ and gold (Au).^[^ ^77^, ^78^, ^79^, ^80^, ^81^ ^]^ The heart issue in the epitaxy is to regulate the orientations of the 2D domains which is closely related the interplay between 2D domains and the single‐crystal substrates.^[^ ^82^, ^83^ ^]^ In consideration of this, we have reviewed and summarized four epitaxial growth modes of 2D materials on different substrates based on their characteristic interactions, namely, van der Waals (vdW) epitaxy, edge epitaxy, step‐guided epitaxy and in‐plane epitaxy (**Figure** **1**). By focusing on the growth mechanism, we hope to gain a deeper insight into the epitaxial growth of 2D materials and explore the potential opportunities to realize large‐scale production of high‐quality 2D single‐crystal materials.

## 2 Fundamentals of 2D Materials Epitaxy

**Figure 2:**
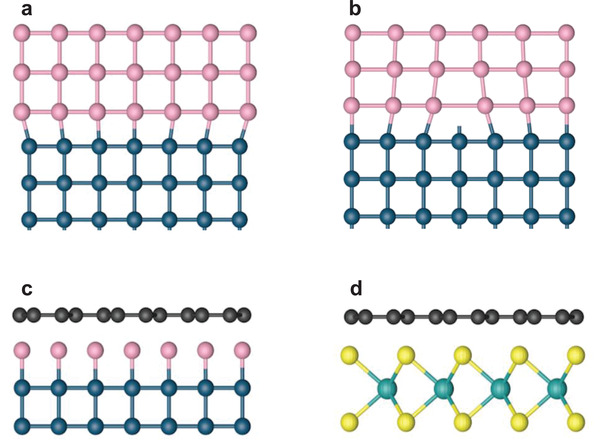
Comparison between conventional epitaxy and vdW epitaxy. Schematics of conventional epitaxy with coherently bonded interface when the lattice match is small a) and with misfit dislocation formation when the lattice mismatch is larger b). c,d) Schematics of vdW epitaxy of 2D materials on dangling‐bond free surface, requiring no lattice match.

Epitaxy on single‐crystal substrates provides a feasible approach to accurately control the domain orientation of epitaxial materials to accomplish their large‐area single‐crystal growth. In the epitaxy of traditional three‐dimensional (3D) materials, the epitaxial layer interacts covalently with the substrate due to the exist of dangling bonds on the surfaces, and strong chemical bonding forms at the interface, which determines the orientation of the epitaxial layer.^[^ ^84^ ^]^ The epitaxy can be attained in systems where the substrate and the epitaxial layer are of the same materials or different from each other. The former is called homoepitaxy while the latter is heteroepitaxy. In the heteroepitaxy, the lattice mismatch between the substrate and epitaxial layer leads to the strain in epilayer or the misfit dislocations at the interface, which may degrade the quality of the epilayer (**Figure** **2**a,b).^[^ ^85^ ^]^ Besides, if the lattice mismatch is too large, the heteroepitaxy is unachievable. In general, lattice mismatch smaller than of 5–10% is required to achieve conventional epitaxy.^[^ ^86^ ^]^ Hence, the rigid lattice matching demand in traditional 3D materials epitaxy extremely restricts the viable material combinations in the heteroepitaxy.

In 1984, Koma et al. first proved the epitaxial growth of Se on cleaved bulk Te crystals and NbSe_2_ films on MoS_2_, which was named as “van der Waals epitaxy.”^[^ ^87^ ^]^ In comparison to conventional epitaxy, the vdW epitaxy features vdW interaction between the substrate and the epilayer, resulting from the vdW interface without surface dangling bonds. So the epitaxy can be realized in bulk materials of which surface dangling bonds have been passivated or in vdW materials which naturally have completely terminated surfaces (Figure 2c,d). The lack of strong covalent bonds at vdW interface significantly reduces the rigorous requirements for lattice matching in conventional epitaxy (the lattice mismatch could be as large as 50% in vdW epitaxy^[^ ^88^ ^]^), which extremely extends heteroepitaxial systems. Meantime, with the rise of 2D materials, the vdW epitaxy has attracted wider attention in the direction of 2D single crystal preparation. It is noteworthy the single‐crystal 2D materials, serving as vdW substrates, also provide new opportunities for the fabrication of many functional materials, including the III‐V compound semiconductors,^[^ ^89^, ^90^ ^]^ metals,^[^ ^91^, ^92^ ^]^ macromolecular polymers,^[^ ^93^, ^94^ ^]^ and so on.

The epitaxy of 2D materials has been achieved on various single‐crystal substrate, but the nuances of epitaxy mechanism on different substrates have yet been discussed carefully. Therefore, the comprehensive understanding of the mechanisms for different 2D materials epitaxy on various types of substrates will be of great value to guide the experimental exploration of controllable synthesis of large‐scale high‐quality single‐crystal 2D materials.

## 3 Epitaxial Growth of 2D Materials

Since the first unambiguously production and identification of graphene in 2004, a large amount of 2D materials have been successfully synthesized in the lab. In the aspect of epitaxial growth of single crystal, the most representative and widely studied 2D materials are semimetal graphene, semiconductor TMDC and isolator h‐BN. These three kinds of 2D materials have also been recognized as the most potential candidates for the practical applications. Besides, they have different lattice structures and their epitaxial growth requires distinct substrates to realize the metal‐catalyzed and noncatalytic epitaxy. By studying their epitaxial growth, we hope to gain a universal understanding of the epitaxy mechanism of different 2D single crystals. Thereby, in this review, we mainly focus on the epitaxial growth of graphene, h‐BN and TMDC.

### 3.1 Van der Waals epitaxy

**Figure 3:**
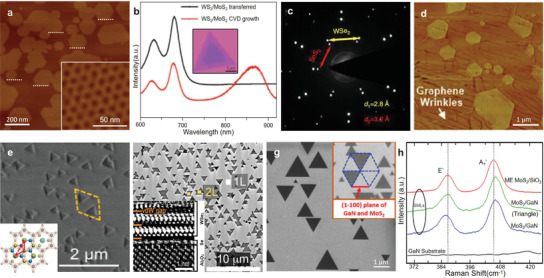
Van der Waals epitaxy. a) Atomic force microscope (AFM) images of aligned hexagonal graphene domains on h‐BN. Inset: Moiré pattern of graphene on h‐BN, demonstrating the alignment between graphene and h‐BN. Reproduced with permission.^[^ ^95^ ^]^ Copyright 2013, Nature Publishing Group. b) PL spectra of a CVD‐grown vertically stacked WS_2_/MoS_2_ bilayer and a transferred one. Inset: Optical image of the CVD‐grown WS_2_/MoS_2_ heterostructures with MoS_2_ showing a light purple color. Reproduced with permission.^[^ ^96^ ^]^ Copyright 2014, Nature Publishing Group. c) SAED patterns of WSe_2_/SnS_2_ heterostructure showing their aligned lattice. Reproduced with permission.^[^ ^97^ ^]^ Copyright 2017, Nature Publishing Group. d) AFM image of hexagonal MoS_2_ domains on CVD‐grown graphene films on Cu. Reproduced with permission.^[^ ^98^ ^]^ Copyright 2012, American Chemical Society. e) Scanning electron microscope (SEM) images of MoS_2_ on mica with two preferential orientations of either 0° or 60°. Inset: Schematic illustration of epitaxial relationship between MoS_2_ and mica. Reproduced with permission.^[^ ^75^ ^]^ Copyright 2013, American Chemical Society. f) Epitaxy of WSe_2_ on *c*‐plane sapphire with surface passivated by Se which can be clearly figured out in the cross‐section scanning transmission electron microscopy (STEM) image (insert). Reproduced with permission.^[^ ^108^ ^]^ Copyright 2018, American Chemical Society. SEM image g) and Raman spectra h) of MoS_2_ domains epitaxially grown on GaN. Reproduced with permission.^[^ ^111^ ^]^ Copyright 2016, American Chemical Society.

Van der Waals epitaxy, as the name suggests, is modulated by the vdW interaction between the 2D material and substrate, which requires that the substrate surface is free of dangling bonds. In view of this, 2D materials are perfect substrates for vdW epitaxy, and the stacking structure of 2D materials is called vdW heterostructure. With great efforts, various vdW heterostructures have been successfully grown.^[^ ^95^, ^96^, ^97^, ^98^, ^99^, ^100^, ^101^, ^102^, ^103^, ^104^ ^]^ Graphene/h‐BN heterostructure was first directly grown by chemical vapor deposition (CVD) method in 2013 and many exotic properties have been observed in this heterostructure due to the formation of the Moiré pattern.^[^ ^95^ ^]^ **Figure** **3**a shows well aligned graphene domains epitaxially grown on the h‐BN substrate, producing the moiré pattern with a periodicity of 15 ± 1 nm, by which we can know that the lattice directions of graphene and h‐BN are parallel.^[^ ^95^ ^]^ Similarly, in a WS_2_/MoS_2_ heterostructure prepared by vdW epitaxial growth, the edges of triangles are parallel with each other and an extra photoluminescence (PL) peak resulting from an interlayer excitonic transition can be observed (Figure 3b),^[^ ^96^ ^]^ indicating a clean interface which can hardly achieved by mechanical transfer technique. VdW epitaxy can be achieved not only in vdW heterostructures consisting of 2D materials with similar lattice constants and structures, but also in the ones with large lattice mismatch or even different lattice structures.^[^ ^97^, ^98^, ^99^, ^100^, ^101^, ^102^ ^]^ The WSe_2_/SnS_2_ heterostructure with lattice mismatch of 14.3% was successfully grown by a two‐step CVD method and their corresponding selected area electron diffraction (SAED) patterns show two parallel hexagonal diffraction patterns with the calculated lattice spacings consistent with the SnS_2_ (0.32 nm) and WSe_2_ (0.28 nm) (Figure 3c), respectively, also denoting the strain in the epitaxial layer is negligible by forming the commensurate structure of 7 × 7 SnS_2_ on 8 × 8 WSe_2_.^[^ ^97^ ^]^ It has been proved that the vdW epitaxial growth of 2D materials is still valid even for a lattice mismatch as high as 50%.^[^ ^88^ ^]^ TMDC monolayers, possessing three‐atomic‐layer structure with transition metal layer sandwiched between two chalcogen layers, are distinct from graphene and h‐BN, while their heterostructures can also be prepared by vdW epitaxy, such as TMDC/graphene,^[^ ^98^ ^]^ TMDC/h‐BN.^[^ ^99^, ^100^, ^101^, ^102^ ^]^ Shi et al. achieved a direct growth of MoS_2_ domains on CVD‐grown graphene film by vdW epitaxy (Figure 3d), which have the same lattice orientation with underneath graphene film.^[^ ^98^ ^]^ But, we should note here that the aligned hexagonal MoS_2_ domains actually have two orientations with 60° rotation, also named inversion orientations, which are energetically equivalent, but different in lattice structure. And when the neighboring two domains with inversion orientations merge together, twin boundary will form.^[^ ^105^, ^106^ ^]^

VdW epitaxial growth can also be achieved on some inactive bulk substrates, such as mica, sapphire, gallium nitride (GaN) and so on. Fluorphlogopite mica (KMg_3_AlSi_3_O_10_F_2_) with layered structure is acknowledged to be an excellent vdW epitaxy substrate for growing 2D materials, because it has atomic flatness, surface inertness and no dangling bonds. Centimeter‐scale uniform monolayer MoS_2_ on mica has been successfully synthesized with two preferential orientations of either 0° or 60° (Figure 3e).^[^ ^75^ ^]^ It is noteworthy that the epitaxial relation was built by the rotation of MoS_2_ lattice by 30° where the lattice mismatch was greatly reduced to only −2.7%, as the schematics shown in the insert in Figure 3e. Moreover, the vdW epitaxy also occurs when the surface of bulk substrate is passivated.^[^ ^107^, ^108^, ^109^ ^]^ Sapphire is a thermally stable substrate compatible with the harsh environments required for CVD growth of TMDC. Figure 3f displays WSe_2_ domains epitaxially grown on the Se‐passivated *c*‐plane sapphire which also possess two preferential orientations.^[^ ^108^ ^]^ And the commensurability between WSe_2_(0.328 nm) and sapphire (0.476 nm) can be 3 × 3 WSe_2_ on 2 × 2 *c*‐plane sapphire.^[^ ^106^, ^110^ ^]^ Similarly, h‐BN could be successfully epitaxially grown on Ge(100) and Ge(110) surfaces passivated by nitrogen.^[^ ^109^ ^]^ Actually, even if the surface is not passivated, the interaction between the substrate and the 2D materials is also the vdW interaction. To distinguish from the epitaxy on dangling bond free substrate, we call the epitaxial growth of 2D materials on a surface with dangling bonds as quasi‐vdW epitaxy. For example, MoS_2_ can be grown on GaN(0001) with MoS_2_ domain edges aligned with the ($1\bar{1}00$) plane of GaN (Figure 3g),^[^ ^111^ ^]^ confirming the epitaxial relation between GaN and MoS_2_ lattices. Compared with the Raman spectrum of mechanically exfoliated monolayer MoS_2_ on SiO_2_/Si substrate, peak position for the strain‐sensitive in‐plane mode $E_{2g}^{1}$ is almost the same for MoS_2_ directly grown on GaN, indicating neglectable strain during the growth, while the 1 cm^–1^ blue shift of the A_1g_ peak together with no observable change in the position of $E_{2g}^{1}$ is suggestive of a strengthening vdW interaction between MoS_2_ and GaN (Figure 3h).^[^ ^111^ ^]^ That means the interaction between the 2D material and the GaN substrate is enhanced by the dangling bonds, but no chemical bonds are formed. The different energy barriers for precursors absorbed onto dangling‐bond and the dangling‐bond free surfaces make the growth of uniform 2D materials monolayers more feasible in quasi vdW epitaxy. The most common quasi‐vdW epitaxial growth system is virtually the TMDC on *c*‐plane sapphire, namely Al_2_O_3_(0001). The threefold symmetry of 2H‐phase TMDC and its long‐range commensurability with Al_2_O_3_(0001) result in an epitaxial orientation of TMDC domains that is either 0° or 60°.^[^ ^67^, ^68^, ^69^ ^]^ And both the theoretical calculation and optical characterization reveal the nature of vdW interaction between the TMDC and the sapphire substrates with dangling bonds.

In the traditional epitaxy the lattice orientation of epilayer is modulated by the strong chemical bonding between the epilayer and the lattice‐matched substrate, and the lattice orientation of the epilayer is determined at the very beginning of the epitaxial growth. Markedly different from traditional epitaxy, the initially formed nuclei of 2D materials have random orientations in the vdW epitaxy due to the absence of strong chemical bonds.^[^ ^83^ ^]^ On the other hand, the weak vdW interaction between 2D materials and the substrate allows the small nuclei to rotate/translate a certain degree.^[^ ^112^ ^]^ Theoretical calculations have revealed that the binding energy between the 2D materials and substrate varied with the rotation angles and the small nuclei could rotate till to the most stable orientation at the growth condition.^[^ ^83^ ^]^ Hence, the epitaxial relation can be established by the nuclei rotation driven by the vdW interaction between 2D materials and the substrate. Although the nuclei rotation during the growth is only supposed theoretically, the rotations of small flakes of 2D materials on vdW surfaces have been widely observed.^[^ ^113^, ^114^ ^]^

**Figure 4:**
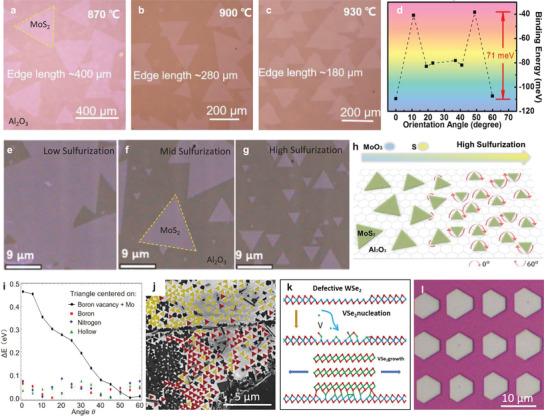
Modulations of van der Waals epitaxy. a–c) Optical images of MoS_2_ grown on sapphire at different growth temperature. Reproduced with permission.^[^ ^70^ ^]^ Copyright 2020, American Chemical Society. d) Calculated binding energy as a function of the relative orientation between MoS_2_ and *c*‐plane sapphire. Reproduced with permission.^[^ ^115^ ^]^ Copyright 2015, American Chemical Society. Optical images e–g) and schematic illustration h) of MoS_2_ domain orientations evolution as the sulfur to molybdenum ratio increases. Reproduced with permission.^[^ ^116^ ^]^ Copyright 2017, American Chemical Society. i) Total binding energies (relative to the ground state for each case) of finite MoS_2_ flakes on monolayer h‐BN with a boron vacancy and Mo interstitial as a function of stacking angle between MoS_2_ and h‐BN. j) SEM image of triangular MoS_2_ flakes on h‐BN. Reproduced with permission.^[^ ^121^ ^]^ Copyright 2019, American Physical Society. k) Illustration of the nucleation and growth of VSe_2_ on the W‐terminated surface at the patterned sites. l) Optical image of periodic arrangements of VSe_2_/WSe_2_ vdW heterostructure arrays. Reproduced with permission.^[^ ^123^ ^]^ Copyright 2020, Nature Publishing Group.

Many studies have been carried out to explore the efficient way to modulate vdW epitaxy. By changing growth temperature, it was found that relatively high growth temperatures were conducive to obtain well aligned MoS_2_ domains on Al_2_O_3_(0001) (**Figure** **4**a–c).^[^ ^70^ ^]^ Combined with theoretical calculations, the MoS_2_ domains with preferential orientations were demonstrated to have maximum binding energy with Al_2_O_3_(0001) (Figure 4d).^[^ ^115^ ^]^ It also means that the coupling between the 2D material and the substrate is stronger at the preferential orientations, in accordance with experimental observations that the photoluminescence signals from the aligned TMDC domains on sapphire were strongly suppressed compared with that of the randomly oriented ones owing to the strain induced by strong coupling.^[^ ^69^ ^]^ Experimentally, it was explained that as the temperature increases the thermodynamic energy (*k*_B_ *T*) could overcome the rotational barrier of the small nucleus to achieve the preferential orientation.^[^ ^67^, ^115^ ^]^ Many works have demonstrated the synthesis of high‐quality films with well‐aligned domains at relatively high temperature, whereas too high temperature can induce dispersed orientations of the initial nuclei where the binding energy differences for different orientations could possibly be smeared out by the thermodynamic energy.^[^ ^68^, ^108^ ^]^

In practice, the epitaxial growth of MoS_2_ can also be realized at relatively low temperature. Aljarb et al. reported that at the growth temperature of 750 °C smaller well‐aligned MoS_2_ domains can be grew on *c*‐plane sapphire substrate with high concentration of S feeding, while larger random oriented ones were obtained with low concentration of S feeding (Figure 4e–h).^[^ ^116^ ^]^ Similarly, high Se/W ratio contributed to the alignment growth of WSe_2_ on *c*‐plane sapphire.^[^ ^117^ ^]^ In the CVD growth of TMDC, metal precursors are always in the solid state. If the metal‐containing particles are not fully reduced during the growth, they would serve as the nucleation sites and the nuclei lose their rotational freedom, resulting in failure of vdW epitaxy.^[^ ^118^ ^]^ Hence, adequate reduction of metal precursors may facilitate the vdW epitaxy. Now it can be understood that high ratio of chalcogen and transition metals ensures sufficient reduction of metal precursors and thus the domains alignment. Besides, high chalcogen evaporation temperatures and early feeding of chalcogen vapors are also conducive to achieve the alignment of TMDC domains.^[^ ^116^, ^117^ ^]^ It also should be noted that considering the smaller nucleus has lower barrier to rotate, the growth rate of nucleus should be as slow as possible that it has enough time to rotate to the preferred orientation.^[^ ^116^, ^117^ ^]^ To sum up, from the perspective of precursor reaction, sufficient reduction of metal precursors and appropriate reaction rate are favorable for vdW epitaxy. Meantime theoretical calculation results revealed that TMDC domain alignment on sapphire with Al‐terminated surface is more feasible due to higher absorption energy.^[^ ^119^ ^]^ In the experiments, early feeding of sulfur and hydrogen at high temperature can reduce sapphire surface by removing oxygen, resulting in an Al‐terminated surface and further better domains alignment.^[^ ^69^, ^119^, ^120^ ^]^

VdW epitaxial growth of 2D materials can achieve well‐aligned domains and thus improve film quality after the domains merging with each other. However, the vdW epitaxy of h‐BN and TMDC whose lattices have threefold symmetry always results in antiparallel domains and thus the twin boundaries when the domains stitching with each other, which may degrade the film performance.^[^ ^115^, ^119^ ^]^ The antiparallel domains formation is due to the binding energy degeneracy and only when it is broken can the unidirectionally aligned domains be grown. According to density functional theory (DFT) results (Figure 4i), the binding energy differences between two inversion orientations of MoS_2_ triangles on h‐BN with interstitial B vacancy and Mo adatom at the center can reach 0.5 eV, totally different from the much weaker variation in the binding energy of the same MoS_2_ flake on h‐BN without defects, where the center of the flake lies above a B atom (red squares), N atom (blue diamonds), or hollow site (green triangles) as the flake is rotated.^[^ ^121^ ^]^ This defect‐induced orientational preference has also been demonstrated experimentally. As show in Figure 4j, ∼90% of the MoS_2_ domains grown on h‐BN by physical vapor transport (PVT) method that prioritizes the initial heterogeneous nucleation of metal species at the boron vacancy have the same orientation.^[^ ^121^ ^]^ The authors also realized the orientation as well nucleation density control by the h‐BN surface defects density and achieved well aligned WSe_2_ domains with a single preferred orientation (84%) using metal organic CVD (MOCVD) method.^[^ ^122^ ^]^ Similarly, dot defects with W dangling bonds produced by a focused laser irradiation can induce a fully oriented VSe_2_ nucleation and growth on the WSe_2_ (Figure 4k,l).^[^ ^123^ ^]^ The introduction of point or dot defects to the single‐crystal substrate can not only contribute to the controllable nucleation of 2D materials, but also modulate their interaction with the substrate and further realize the unidirectional epitaxial growth. This strategy may provide an efficient way to prepare large‐area single‐crystal vertical stacking heterostructures.

The lack of strong covalent bonds at vdW interface significantly reduces the rigorous requirements for lattice matching in conventional epitaxy, which extremely extends heteroepitaxial systems. However, due to the weak van der Waals interactions, multiple preferential orientations are easily formed in the growth of low‐symmetry 2D materials, such as TMDC materials with threefold symmetry.^[^ ^106^ ^]^ In addition, no dangling bonds will lead to difficulties in nucleation of 2D domains on the substrate.

### 3.2 Edge Epitaxy

The surface of 2D materials is free of dangling bonds, while there are inevitable dangling bonds at the edge. When growing 2D materials on some chemically active substrates, such as metal substrate, which serves as both catalyst and substrate to promote the dissociation of precursors and the synthesis of 2D materials,^[^ ^124^ ^]^ the edge atoms of 2D materials will interact with the metal substrate strongly and form chemical bonds.^[^ ^125^, ^126^, ^127^, ^128^, ^129^, ^130^, ^131^, ^132^ ^]^ In the nucleation stage, the number of atoms in the domain varies from a few to a few dozen,^[^ ^126^ ^]^ and the proportion of edge atoms is relatively large. Therefore, the chemical interaction between the edge atoms and the substrate is much stronger than the intralayer vdW interaction,^[^ ^127^ ^]^ and the orientation of nucleus is determined by the edge interactions. In this case, the epitaxial growth is called “edge epitaxy.”^[^ ^127^ ^]^

**Figure 5:**
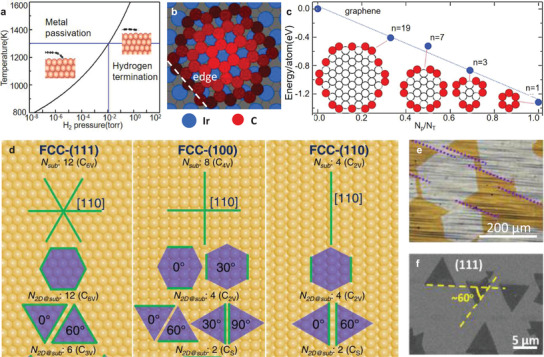
Edge epitaxial growth of 2D materials. a) Diagram of graphene zigzag edge passivation on the Cu(111) surface at different hydrogen pressures and growth temperatures. Reproduced with permission.^[^ ^133^ ^]^ Copyright 2014, American Chemical Society. b) Calculated structural models of the dome‐shaped C clusters formed on Ir(111). c) Evolution of the binding energy per atom as a function of the ratio of C atoms at the periphery (*N*_P_) and the total number (*N*_T_) in each C cluster. n indicates the number of honeycomb rings. Reproduced with permission.^[^ ^125^ ^]^ Copyright 2009, American Physical Society. d) Alignment of single‐crystal 2D islands with various symmetries on low‐index face‐centered cubic (FCC) surfaces. Reproduced with permission.^[^ ^140^ ^]^ Copyright 2020, Nature Publishing Group. e) Optical image of aligned graphene domains on single‐crystal Cu(111) with the dashed line indicating the domain edges. Reproduced with permission.^[^ ^44^ ^]^ Copyright 2017, Elsevier. f) SEM image of antiparallel h‐BN domains grown on Cu (111). Reproduced with permission.^[^ ^46^ ^]^ Copyright 2015, Nature Publishing Group.

The most typical edge epitaxial growth systems are the growth of graphene and h‐BN on metal substrates. It is worth mentioning that the edge of the graphene or h‐BN can also be passivated by active atoms from the environment of its growth, such as H or OH groups, where there is only the weak vdW interaction between the 2D material and the substrate, and the law to control the 2D domains orientations follows the vdW epitaxy as discussed in Section 3.1.^[^ ^133^, ^134^ ^]^ The theory calculation results give the conditions for the edge C atoms of graphene being passivated by hydrogen on Cu(111), which are determined jointly by the growth temperature and H_2_ partial pressure (**Figure** **5**a).^[^ ^133^ ^]^ The conditions for hydrogen passivation of 2D edges are varied with different metals.^[^ ^134^ ^]^ Therefore, only under proper growth conditions and on proper substrates the edge epitaxy can be realized. Although the interfacial interactions between graphene and different metal substrates change significantly, the unsaturated graphene edge can always bend to the metal surface and form chemical bonds during the growth.^[^ ^125^, ^126^, ^127^, ^128^, ^129^, ^130^ ^]^ Given this interaction of their edges, dome‐shaped carbon nanoislands would form on the metal surface which can be regarded as the intermediate between carbide clusters and quasi‐freestanding graphene.^[^ ^125^ ^]^ As shown in Figure 5b is an atomic model of a carbon dome on Ir(111).^[^ ^125^ ^]^ Consequently, the interactions between graphene and metals consist of strong chemical bonding at the edges and weak vdW forces between the layers. According to the correlation between the bonding interaction of the cluster and the number of atoms at the periphery, the effect of edge interactions on binding energy decreases with cluster size (Figure 5c).^[^ ^125^ ^]^ Thus at the infancy of growth the orientations of domains are determined by edge interactions and such orientations can be inherited by the matured graphene due to the higher rotation barrier of larger graphene domains. Similar to graphene on metal surface, the strong interactions between the h‐BN edges and underlying metal atoms also lead to the formation of dome‐like geometries.^[^ ^61^, ^132^ ^]^

To elucidate how edge interactions determine the orientation of graphene and h‐BN on metal surface, we focus on their edge epitaxial growth on low‐index single‐crystal Cu substrates, namely Cu(100), Cu(110), and Cu(111). Graphene and h‐BN have the same lattice structures and similar lattice constants (graphene: 2.46 Å; h‐BN: 2.50 Å) and thus exhibit almost the same growth behaviors in the CVD growth. By theoretical calculation, it has been demonstrated that the zigzag (ZZ) edge of graphene and the nitrogen terminated ZZ (N‐ZZ) edge of h‐BN are generally the slowest propagating edges because of their highest propagation barriers.^[^ ^135^, ^136^, ^137^, ^138^, ^139^ ^]^ Graphene has a C_6V_ symmetry and the single‐crystal graphene domain always is perfect hexagonal with ZZ edges, while h‐BN has a C_3V_ symmetry and the single‐crystal h‐BN domain always is triangle with N‐ZZ edges. The ZZ (N‐ZZ) edge of graphene (h‐BN) tends to bond with Cu surface and when it is along the Cu<110> direction, the binding energy has the maximum.^[^ ^140^ ^]^ The strongest bonding of the ZZ (N‐ZZ) edge of graphene (h‐BN) along the Cu <110> direction mainly lies in two aspects: i) The atoms are close‐packed (with high symmetry) and thus the electron density fluctuation is lowest with alternative ridges and valleys of uniform height along <110> direction, leading to the preferential formation of a straight edge. ii) The lattice mismatch is minimum along <110> direction. The <110> direction varies depending on the Cu crystal surface and thus the alignment of graphene and h‐BN is different on three low‐index Cu surface (Figure 5d).^[^ ^140^ ^]^ Theoretically, unidirectional alignment of graphene domains can be achieved on both Cu(111) and Cu(110), while two identical orientations along perpendicular directions on Cu(100). Experimental observations are consistent with this theoretical prediction ideally.^[^ ^44^, ^141^, ^142^ ^]^ Due to a relatively low surface energy, large‐size single‐crystal Cu(111) can be achieved by abnormal grain growth from commercial polycrystal Cu and thus the most feasible synthesis method of large graphene single crystal is to epitaxially grow graphene on Cu(111) currently.^[^ ^44^ ^]^ Figure 5e is an optical image of aligned graphene domains on single‐crystal Cu(111) substrate.^[^ ^44^ ^]^ Actually, above alignment theory is also applicable to the epitaxy of graphene on other FCC metal substrate. For example, graphene grows with a single orientation on the Ge(110) substrate with graphene edges along <110> direction resulting from the Ge‐C covalent bonds at the early stage of growth.^[^ ^53^ ^]^

The edge epitaxy of h‐BN follows the same alignment rules as that of graphene. However, due to the lower symmetry of h‐BN (C_3V_), the inversion triangle h‐BN domains (of 60° rotation) with degenerated energy have different crystallographic directions, and when they merge together twin boundaries would form. Therefore, even on Cu(111) and Cu(110) single‐crystal h‐BN film can hardly be achieved and the domains on Cu(100) have four different orientations.^[^ ^45^, ^46^, ^47^ ^]^ Although the energy degeneration can be broken when the influences from subsurface Cu layer are also taken into consideration, the energy difference of two orientations are so small (several meV) that both orientations appear during the high‐temperature CVD process (Figure 5f, inversion h‐BN domains with 60° rotation grown on Cu(111) substrate).^[^ ^46^ ^]^

In addition, that the slowest propagating edges of a 2D material tend to align along high symmetry directions of the substrate also appears in the vdW epitaxial mode, such as the ZZ edge of TMDC domains along <11$\bar{2}$0> or <1$\bar{1}$00> direction of Al_2_O_3_(0001),^[^ ^67^, ^68^, ^69^ ^]^ <1$\bar{1}$00> direction of Al_2_O_3_(11$\bar{2}$0)^[^ ^71^ ^]^ and <1$\bar{1}$00> direction of GaN (*m*‐plane).^[^ ^111^, ^143^ ^]^ This means the domain alignment rule in edge epitaxy also works for vdW epitaxy. That is to say, no matter the edge is passivated by functional groups in the growth environment or by the metal surface, 2D domains would have the same orientation distribution theoretically on metal substrate.^[^ ^46^, ^47^, ^48^ ^]^ On the other hand, due to the strong chemical bonding between the edge and the metal surface, the edge epitaxial growth of 2D materials on the metal substrate is more robust compared with the vdW epitaxy.

### 3.3 Step‐Guided Epitaxy

**Figure 6:**
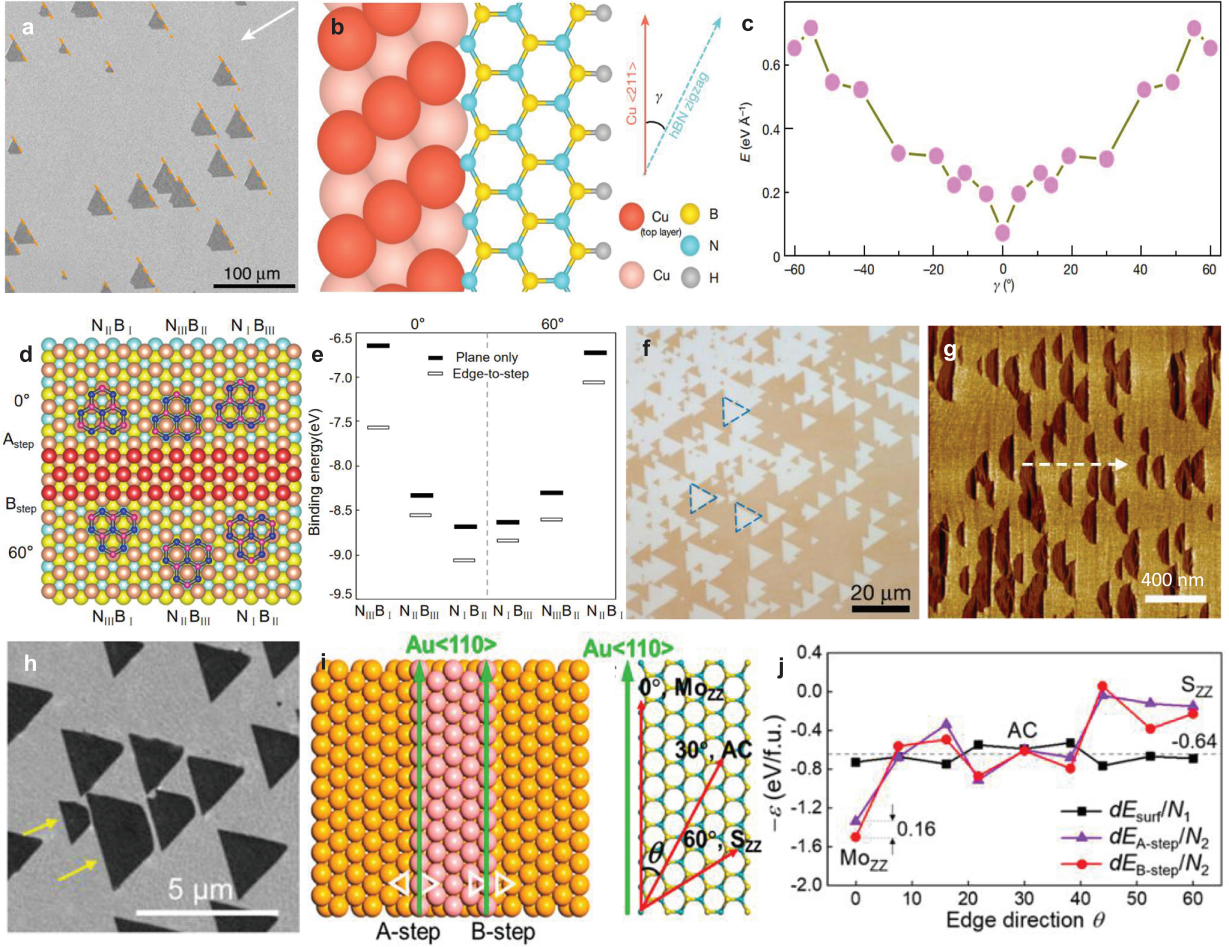
Step‐guided epitaxy of h‐BN, graphene, and TMDCs on metal substrate. a–c) Unidirectionally aligned h‐BN domains on the vicinal Cu(110) substrate. The configuration with the N‐ZZ edge of h‐BN domains bonding with <211> step edge b) has the minimum formation energy c). Reproduced with permission.^[^ ^49^ ^]^ Copyright 2019, Nature Publishing Group. d‐f) Mono‐oriented h‐BN domains on single‐crystal Cu (111). The steps trending up and down (A_step_ and B_step_) d) could lift off the energy differences of the most stable stacking configurations N_I_B_II_ and N_I_B_III_ e) and thus lead to unidirectional alignment of h‐BN on Cu(111) f). Reproduced with permission.^[^ ^52^ ^]^ Copyright 2020, Nature Publishing Group. g) AFM friction image of graphene nanoribbons grown on vicinal Ge(001) surfaces with 12° miscutting angle, with dashed white arrows representing the miscut directions. Reproduced with permission.^[^ ^146^ ^]^ Copyright 2019, Wiley‐VCH. h–j) Unidirectionally aligned MoS_2_ domains on the Au(111) surface. The calculated binding energies (*ε*) of the MoS_2_ nanoribbons with various edge directions bonding to <110> step edges (A‐step and B‐step) or on the surface of the Au(111) substrate reveal that Mo‐ZZ edge bonding to B‐step has the maximum binding energy, which is almost twice of that on the surface, demonstrating the step guidance is dominant in the unidirectional nucleation and growth of MoS_2_. The abbreviation “f.u.” in j) denotes the formula unit. Reproduced with permission.^[^ ^79^ ^]^ Copyright 2020, American Chemical Society.

The aligned growth of 2D materials on different single‐crystal substrates can be realized by vdW or edge epitaxy. Nevertheless, only graphene with sixfold lattice symmetry can have unidirectional domains, which can further seamlessly stitch into single‐crystal films. Most of other 2D materials, including h‐BN and TMDC, have lower symmetry and thus always have antiparallel domains, resulting in twin boundaries which greatly degrade the electronic properties on most substrates.^[^ ^70^, ^119^ ^]^ Tremendous efforts have been made to obtain unidirectional alignment of h‐BN and TMDC. In 2019, Wang et al. successfully demonstrated the ∼99% unidirectional alignment of h‐BN domains on single‐crystal vicinal Cu(110) substrate with Cu step edges along <211> direction (**Figure** **6**a).^[^ ^49^ ^]^ Unlike previous reports, the unidirectional alignment clearly reveals the presence of one and only configuration of h‐BN/vicinal Cu(110). The DFT calculation revealed that the N‐ZZ edge of h‐BN domains bonding with <211> step edge is the exclusive minimum‐energy state (Figure 6b,c).^[^ ^49^ ^]^ This suggests that the inversion degeneracy of h‐BN domains can be lifted by the atomic <211> step on the vicinal Cu(110) surface.^[^ ^144^ ^]^ Combining the experimental observation and theoretical calculation, the “step‐guided epitaxy” was first proposed definitely that the steps edge along the specific direction would guide the unidirectional alignment of 2D materials. In the wake of this work step‐guide epitaxial growth of different 2D materials on different substrates has been springing up.^[^ ^145^ ^]^

On a vicinal low‐index surface or high‐index surface, the steps trend only up or down across the entire surface, while on a low‐index surface, for example, Cu(111) surface, the steps can trend both up and down (Figure 6d).^[^ ^52^ ^]^ It is plausible that the edge‐guided h‐BN domains on such low‐index surface can have both orientations of 0° and 60°. The DFT calculation of binding energy for 6 kinds of stacking configuration of h‐BN cluster on Cu(111) surface demonstrates the existence of steps amplifies the bonding energy difference between the two most stable configurations (N_I_B_II_ and N_I_B_III_), enabling easily achieving the unidirectional alignment of the h‐BN lattice (Figure 6d,e).^[^ ^52^ ^]^ Virtually the experimental observations are well consistent with the theoretical calculation that unidirectionally aligned h‐BN domains can be obtained on a wafer‐scale single‐crystal Cu(111) substrate (Figure 6f).^[^ ^52^ ^]^ Additionally, the step‐guided epitaxy is also applicable to the unidirectional growth of graphene. Figure 6g displays the unidirectionally aligned graphene nanoislands on the 12° miscut Ge(001) surface.^[^ ^146^ ^]^

The step‐guided epitaxy of single‐crystal 2D materials is universal and can also realize the growth of unidirectional domains of TMDC materials. Because Cu is highly reactive with chalcogen atoms,^[^ ^147^ ^]^ growing TMDC materials generally employs the Au substrate in view of its chemical inertness toward the chalcogen precursor in the traditional CVD process.^[^ ^77^, ^148^ ^]^ In 2020, Yang et al. first proposed the step‐guided epitaxial growth of MoS_2_ and obtained wafer‐scale single‐crystal MoS_2_ film on the single‐crystal Au(111) which was prepared by the melting and resolidifying technique.^[^ ^79^ ^]^ The MoS_2_ nucleates and aligns at the step edges along <110> direction of Au(111) substrate and then grows up, producing unidirectionally aligned domains (Figure 6h).^[^ ^79^ ^]^ Some domains have trapezoid shapes, characteristic of the step‐guided growth.^[^ ^49^, ^66^ ^]^ Similar to Cu(111), there are both step‐up and step‐down edges along the <110> direction on Au(111), named as A‐step and B‐step in Figure 6i.^[^ ^79^ ^]^ The DFT calculation indicates that Mo terminated ZZ (Mo‐ZZ) edge attaching to B‐step has the minimum contact energy, 0.16 eV/(formula unit) lower than that of Mo‐ZZ edge attaching to A‐step, meaning Mo‐ZZ edge attaching to B‐step is energetically more favorable, which thus facilitates unidirectional growth of MoS_2_ domains (Figure 6j).^[^ ^79^ ^]^ Additionally, the binding energy maximum of step contact is almost twice as that of the surface contact, indicating that the interaction between step and Mo‐ZZ edges plays a decisive role in the oriented nucleation and growth of MoS_2_ domains. In 2021 Choi et al. successfully grew several TMDC single‐crystal monolayers on various high‐index Au surface, demonstrating the universality of this step‐guided epitaxial growth of TMDC.^[^ ^81^ ^]^ It was also confirmed that the orientation of domains is independent of surface index but step direction, consistent with the above analysis on the mechanism of step‐guided growth. This strategy provides a general avenue for the single‐crystal growth of 2D materials.

**Figure 7:**
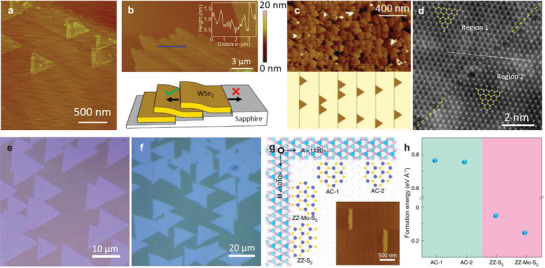
Step‐guided epitaxy of TMDCs on sapphire substrate. Step‐guided aligned nuclei a) and overlapping layers b) of WSe_2_ on *c*‐plane sapphire with atomic steps by CVD growth. Reproduced with permission.^[^ ^66^ ^]^ Copyright 2015, American Chemical Society. AFM c) and STEM image of the stitching region d) of unidirectionally aligned WS_2_ domains grown on *c*‐plane sapphire by MOCVD method. The line defect at the stitching region may be caused by the translational offset between the aligned domains. Reproduced with permission.^[^ ^72^ ^]^ Copyright 2021, American Chemical Society. Optical images of parallel e) and antiparallel f) MoS_2_ domains grown on *c*‐plane sapphire with step along <10$\bar{1}$0> and <11$\bar{2}$0> directions, respectively. Schematic illustration g) and calculated formation energy h) of four possible edge configurations of MoS_2_ nuclei at the <10$\bar{1}$0> and <11$\bar{2}$0> steps on *c*‐plane sapphire, respectively. Inset in g): AFM image of MoS_2_ growing along the <10$\bar{1}$0> step edges. Reproduced with permission.^[^ ^73^ ^]^ Copyright 2021, Nature Publishing Group.

Actually, as early as 2015, the aligned growth of 2D WSe_2_ on *c*‐plane sapphire guided by the steps has been observed (**Figure** **7**a).^[^ ^66^ ^]^ When the growth temperature is above 950 °C, parallel atomic steps along <11$\bar{2}$0> direction are formed due to surface reconstruction of sapphire substrate and can guide the nucleation and growth of aligned WSe_2_ domains.^[^ ^66^ ^]^ However, in their experiments the uphill WSe_2_ domains cannot stitch with the downhill ones but overlap on them when propagating across the step edge (Figure 7b).^[^ ^66^ ^]^ Thus they could not achieve the continuous single‐crystal WSe_2_ film. This year the WS_2_ monolayers on *c*‐plane sapphire with well‐defined <11$\bar{2}$0> steps were synthesized using MOCVD growth.^[^ ^72^ ^]^ By designing a multistep variable‐temperature growth process, high nucleation density and small unidirectionally aligned WS_2_ domains were obtained (Figure 7c).^[^ ^72^ ^]^ In this case, the small domains could stitch with each other, forming a continuous film. However, the STEM image reveals the existence of translational boundaries that may arise from a slight lattice offset between coalescing aligned domains (Figure 7d), which are rarely observed in the single‐crystal films grown on the metal substrate.^[^ ^72^ ^]^ This is because the high stability of the sapphire at the growth temperature makes the surface atoms movement extremely hard and thus it is difficult to offset lattice translational mismatch between neighboring WS_2_ domains by sapphire surface atoms movement, which is a common phenomenon for metal substrates.^[^ ^79^, ^81^ ^]^ Therefore, it requires more careful control of the growth kinetics to achieve perfect domains stitching and further high‐quality single‐crystal films on sapphire substrate. Recently, Li et al. realized the epitaxial growth of wafer‐scale single‐crystal MoS_2_ on *c*‐plane sapphire with steps along <1$\bar{1}$00> direction under S‐rich condition.^[^ ^73^ ^]^ Their experimental results demonstrated that unidirectionally aligned MoS_2_ domains can only be grown on the surface with <1$\bar{1}$00> steps, in contrast with those of inversion orientations on the surface with <11$\bar{2}$0> steps (Figure 7e,f).^[^ ^73^ ^]^ The first‐principles calculation results reveal that under S‐rich condition, the Mo‐ZZ edge with a 100% S coverage (ZZ‐Mo‐S_2_) bonding with <1$\bar{1}$00> step edge is the most stable configuration, with formation energy of 0.1 eV Å^–1^ lower than that of the inversion domain (ZZ‐S_2_), while the two orientations domains bonding with <11$\bar{2}$0> step edge have comparable formation energies (Figure 7g,h).^[^ ^73^ ^]^ Thus it should be noted here that only the steps that can lift the energy degeneracy of inversion orientations could guide the unidirectional alignment of nucleation and growth of the 2D domains. The aligned domains could merge into a continuous single‐crystal film perfectly, demonstrated by both the atomic resolved STEM image and polarized second harmonic generation mapping, and the film exhibits excellent uniformity and electronical performance. Based on above experimental observations and theoretical calculations, we can obtain almost complete understanding toward the step‐guided epitaxial growth. Generally, the step edges on the substrate surfaces could act as preferential nucleation sites and 2D materials tend to nucleate at step edges by the formation of energetically stable chemical bonds with edge atoms.^[^ ^126^, ^145^ ^]^ Thus lattice match between the step edges and 2D edges is needed and the step direction and the 2D edge configuration concurrently determine the alignment of the 2D materials.^[^ ^144^, ^149^ ^]^

**Figure 8:**
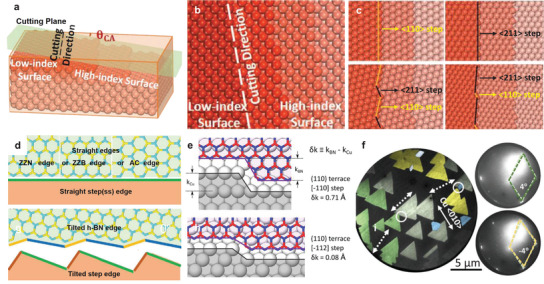
Atomic steps on metal surfaces. a,b) schematic diagram showing cutting of a single crystal with a high‐index surface where *θ*_CA_ denotes the cutting angle. c) Different possible types of step edges on Cu(111) surface. d) Interfaces between h‐BN and step edges. Reproduced with permission.^[^ ^149^ ^]^ Copyright 2021, Wiley‐VCH. e) Complementarity of kinks at the h‐BN edge (red B, blue N) to the steps of the metal surface. *δ*k indicates the mismatch in kink height. Reproduced with permission.^[^ ^144^ ^]^ Copyright 2019, American Chemical Society. f) Color‐false dark field low energy electron microscopy (LEEM) image (left) and low energy electron diffraction (LEED) patterns (right) of h‐BN on Cu. The colors of h‐BN triangles are assigned according to the diffraction patterns, and the white dashed arrows represent the step directions. Reproduced with permission.^[^ ^50^ ^]^ Copyright 2019, Wiley‐VCH.

High‐index surfaces ideally with parallel step along specific directions provide abundant surface structures to investigate the epitaxial growth of 2D materials. Traditionally a high‐index surface can be prepared by cutting a low‐index single crystal along a specific direction. The cutting plane is the high‐index facet with step direction along the line intersecting the low‐index surface and step density is determined by the cutting angle (*θ*_CA_) between the cutting plane and its nearest low‐index facet (**Figure** **8**a,b).^[^ ^149^ ^]^ We take Cu as an example for detailed analysis. According to crystallography step orientations of FCC metal could be along <100>, <110>, and <211> directions. If the cutting direction is along one of them, straight steps will be produced, otherwise tilted steps (step with kinks) may form. For example, Cu(111) serial high‐index surfaces (the one with the terrace of (111) facet) have two types of straight steps along <110> or <211> directions and two types of tilted steps consisting of two different components of straight steps (Figure 8c).^[^ ^149^ ^]^ A straight 2D edge attaching to a straight step edge i) and a tilted 2D edge attaching to a titled step edge ii) are two energetically favorable interfaces (Figure 8d).^[^ ^149^ ^]^ For the case (i), once the lattice match between the step edge and 2D edge is met, unidirectional alignment of 2D domains can be achieved. For the case (ii), the larger step edges would determine the primary direction of the domain, while the matching degree between metal kinks (k) and 2D kinks (*k*_2D_) also affect the primary direction. Only when the kinks complement each other, that is, the offset *δ*_k_ = *k*_Cu_ − *k*_2D_ is small (<0.1 Å), the primary orientation of the domains can be preserved in the presence of the kink (Figure 8e).^[^ ^144^ ^]^

Ideally, the high‐index surface is consisted of parallel and evenly spaced straight or tilted steps, while in the real growth condition, surface roughening is inevitable which would lead to step meandering. This step direction change can also alter the orientation of the attached 2D domains as shown in Figure 8f.^[^ ^50^ ^]^ Theoretical calculations revealed that the high‐index surface with a large cutting angle was more robust to surface roughness and thus to achieve unidirectionally aligned 2D domains.^[^ ^51^, ^144^ ^]^ Additionally, cutting angle also determines the step density and further the epitaxial growth behaviors of 2D domains.^[^ ^149^ ^]^ For instance, graphene nanoribbons of high aspect ratios can be grown on Ge(001) by edge epitaxy and the two dominant orientations with same proportions (perpendicular to each other) were ascribed to the surface atomic reconstruction on the terrace.^[^ ^146^ ^]^ However, the growth behaviors of graphene nanoribbons change as the cutting angle (cutting toward the <111> direction of Ge(001) substrate) increases. With the increment of cutting angle, step density increases so that step‐induced epitaxial growth predominates, resulting in unidirectional growth of nanoribbons when the cutting angle is larger than 10°. Thus by rational design of step direction and step density, it could be robust to achieve the step‐guided epitaxy and further realize the fabrication of high‐quality single‐crystal 2D materials films.

### 3.4 In‐Plane Epitaxy

**Figure 9:**
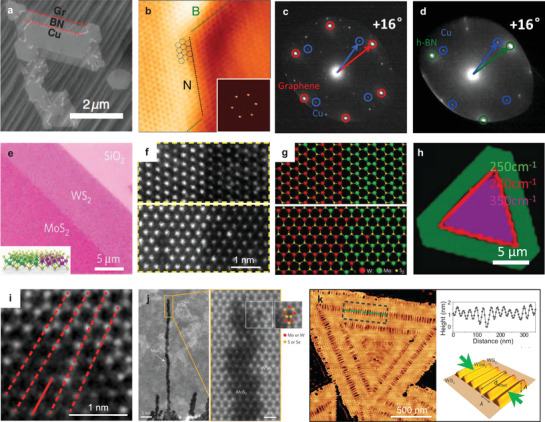
In‐plane epitaxy of 2D materials. a) SEM image of h‐BN epitaxially grown at graphene edges. Reproduced with permission.^[^ ^151^ ^]^ Copyright 2014, Science AAAS. b) STM images of the stitching region of h‐BN/graphene lateral heterostructure with the h‐BN edge terminations marked with dotted lines. Reproduced with permission.^[^ ^152^ ^]^ Copyright 2014, Nature Publishing Group. c,d) LEED patterns of Cu, graphene, and h‐BN marked by blue, red, and green circles, respectively. Reproduced with permission.^[^ ^151^ ^]^ Copyright 2014, Science AAAS. e) Optical image of the WS_2_/MoS_2_ lateral heterostructure on SiO_2_/Si substrate, showing the abrupt contrast change at the interface. Atomic‐resolution STEM images f) and atomic models g) of the atomically sharp lateral interfaces along the zigzag and armchair directions. Reproduced with permission.^[^ ^96^ ^]^ Copyright 2013, Nature Publishing Group. h) Raman mapping of the WS_2_/MoSe_2_/WSe_2_ multi‐heterostructure grown on SiO_2_/Si. Reproduced with permission.^[^ ^161^ ^]^ Copyright 2017, Science AAAS. i) Atomically resolved annular dark‐field (ADF) STEM image of WSe_2_/MoS_2_ lateral heterojunction with solid line indicating the misfit atomic plane. Reproduced with permission.^[^ ^163^ ^]^ Copyright 2018, Nature Publishing Group. j) ADF‐STEM image of MoS_2_ one‐dimensional (1D) channels embedded within WSe_2_. The channel ends with the 5|7 dislocation (white box in right column). Reproduced with permission.^[^ ^171^ ^]^ Copyright 2017, Nature Publishing Group. k) AFM mapping (left) and height profile (upper right) of a representative WS_2_/WSe_2_ superlattice, demonstrating the unique structure with rippled WSe_2_ and flat WS_2_. Reproduced with permission.^[^ ^170^ ^]^ Copyright 2018, Science AAAS.

In‐plane epitaxy means the epitaxial growth of one 2D material from the edge of the other one, forming a lateral heterojunction with covalently bonded interface. Unlike vertical heterojunctions, which can be prepared by physical stacking, lateral heterojunctions can only be achieved by direct growth. In 2012, Levendorf et al. first reported the graphene/h‐BN lateral heterostructure by a patterned regrowth strategy and demonstrated the mechanical continuity of the junction, and the conductivity of graphene and insulativity of h‐BN in the patterned film maintained excellently.^[^ ^150^ ^]^ Liu et al. gave the explicit experimental results to show the epitaxial growth of h‐BN from the edge of graphene domain on Cu substrate. They utilized hydrogen to etch graphene to obtain fresh edges, where h‐BN nucleated and grew (**Figure** **9**a).^[^ ^151^ ^]^ The atomic‐resolved scanning tunneling microscope (STM) image shows the stitching boundaries and lattice coherency between graphene and h‐BN (Figure 9b).^[^ ^152^ ^]^ Also it can be observed that at the stitching boundaries h‐BN has B/N‐zigzag terminations. The h‐BN lattice adopts the orientation of the graphene independent of the underlying Cu lattice as proven by LEED (Figure 9c,d), because the in‐plane bonding is much stronger than and thus overrides the out‐of‐plane vdW interaction with the Cu.^[^ ^151^ ^]^ Also graphene can be epitaxially grown from h‐BN edge.^[^ ^153^, ^154^, ^155^ ^]^ Liu et al. used photolithographically patterned h‐BN monolayers as templates to grow graphene to produce patterned heterojunctions.^[^ ^156^ ^]^ And in‐plane superlattices of alternating graphene and h‐BN could be achieved directly via a CVD process by feeding alternating streams of C and B, N precursor gases.^[^ ^157^ ^]^ The dangling bonds make the nucleation barrier at the 2D edge lower than on the 2D surface, and so nucleation is preferred at the edge under lower growth temperature or lower precursor concentrations.^[^ ^158^, ^159^ ^]^ On the other hand, nucleation rates at edge or on the surface become comparable under high temperature or high precursor concentration.^[^ ^158^ ^]^ Gao et al. developed a temperature‐triggered reaction route in CVD process to achieve the selective growth of h‐BN/graphene lateral and vertical heterostructures on Cu foils.^[^ ^160^ ^]^ Such an atomic‐scale seamless stitching in the h‐BN/graphene lateral heterojunction is because the same lattice structure and small lattice mismatch (1.7%) between graphene and h‐BN, which could be accommodated via in‐plane bond length variations. Besides, that h‐BN and graphene have comparable interactions with the metal substrate, giving a consistent overlayer‐substrate distance and a highly comparable surface corrugation, is of great significance to achieve the perfect stitching during the growth.

In‐plane epitaxy of 2D materials requires the matching of the crystal structure and this is what we associate with conventional epitaxy, where the difference is simply the bonding of 2D and 1D interfaces. The similar lattice structure of TMDC provides a prerequisite for in‐plane epitaxial growth. In 2014, Gong et al. successfully prepared high‐quality in‐plane heterostructures of WS_2_/MoS_2_ in a one‐step growth where the sequential growth of MoS_2_ and WS_2_ was achieved due to their nucleation and growth rates difference (Figure 9e).^[^ ^96^ ^]^ They found at low temperature WS_2_ could epitaxially grow from MoS_2_ edges, resulted from the advantage of low nucleation potential at the edge due to dangling bonds. The high‐resolution STEM images show the atomic structures of heterojunction interface where MoS_2_ and WS_2_ stitch seamlessly into a single hexagonal lattice and share the same orientation (Figure 9f,g).^[^ ^96^ ^]^ Atomically sharp zigzag interface with metal‐terminated and chalcogen‐terminated zigzag edges joined by covalent bonds, was preferred in such heterojunctions. This interface is common in TMDC heterogeneous epitaxy, which means that this interface is the most stable in terms of energy.^[^ ^161^, ^162^, ^163^, ^164^ ^]^ Whereas, armchair interface was also observed with slight interdiffusion of metal elements, possibly due to the low stability of the armchair edge during the growth.^[^ ^96^ ^]^ Meantime Duan et al. designed a CVD system that allowed in situ switching of the solid source (WS_2_ and WSe_2_) into and out of the hot zone to enable the sequential growth of single‐ or few‐layer WS_2_/WSe_2_ lateral heterostructures.^[^ ^165^ ^]^ One‐step growth strategy could guarantee the fresh edge during the growth, which is beneficial to achieve the coherent stitching interface, while it also makes the alloying inevitable, resulting in the inhomogeneous composition and properties of the lateral heterojunction.^[^ ^166^, ^167^ ^]^ To tackle the alloying problems, researchers have developed various techniques. Li et al. applied two‐step growth method where they grew WSe_2_ at a higher temperature first and then grew MoS_2_ in a separate furnace at a lower temperature to avoid any alloy reaction. They also could achieve high‐quality lateral heterojunction with atomically sharp interface.^[^ ^74^ ^]^ Furthermore, as‐grown TMDC monolayers can be patterned and the exposed edges serve as the seeds for growing a second TMDC material to form desired lateral heterostructures with arbitrary layouts.^[^ ^168^, ^169^ ^]^ It is worth mentioning that Zhang et al. introduced a reverse flow during the temperature swing between the successive growth stages to cool down the as‐grown 2D domains to avoid the excessive thermal degradation or uncontrolled nucleation.^[^ ^161^ ^]^ By this trick, they could realize the robust preparation of a wide range of multi‐heterostructures and superlattices with precisely controlled structure and atomically sharp interface. Figure 9h is the Raman mapping of WS_2_/WSe_2_/MoS_2_ multi‐heterostructure.^[^ ^161^ ^]^

Due to the covalent bonding at the interface in in‐plane epitaxy, the difference in lattice constants between the two materials can introduce stress at the interface.^[^ ^163^, ^170^ ^]^ When the lattice mismatch is small, the stress can be released by in‐plane bond length variations (lattice strain), as the case of h‐BN/graphene heterojunction. But when the mismatch is large, dislocation at the interface or out‐of‐plane ripple may form. The ADF‐STEM image of a lateral WSe_2_/MoS_2_ heterojunction (lattice mismatch of 3.8%) with dashed red line indicating the atomic plane clearly shows the formation of misfit dislocation at the interface (Figure 9i).^[^ ^163^ ^]^ More interestingly, Han et al. found the dislocation at the interface of WSe_2_/MoS_2_ could induce the growth of sub‐nanometer‐wide 1D MoS_2_ into the WSe_2_ monolayers and the edge of MoS_2_ is perfectly stitched with WSe_2_, forming a coherent interface (Figure 9j).^[^ ^171^ ^]^ The higher reactivity in the core of the dislocations allows the Mo and S to be inserted into the dislocation core, thus pushing the dislocations away from the original interface, forming 1D MoS_2_ channels. Actually, this dislocation‐guided 1D channel epitaxial growth can not only occur at the heterojunction interface, but also at the grain boundaries of 2D film with a periodic distribution of dislocations. This opens a new path for the synthesis of 1D superlattices in 2D monolayers. In a coherent WSe_2_/WS_2_ superlattice (lattice mismatch of 4%), the compressive strain in WSe_2_ with width smaller than 320 nm could be released by out‐of‐plane rippling, as shown in Figure 9k, while when the width of WSe_2_ is larger than 320 nm, the rippling is not continuous anymore and other strain‐release path may form, such as the misfit dislocations.^[^ ^170^ ^]^ Therefore, tuning the supercell dimensions would engineer the epitaxial strain in the superlattices, and further the performances, which is unique in 2D in‐plane epitaxy.

The lattice match requirement makes the in‐plane epitaxy of 2D materials can only achieved for the ones with same crystal lattice. For example, TMDC materials can also nucleate at the edge of graphene, but only form an overlap junction with graphene rather than a stitching and coherent interface.^[^ ^172^, ^173^ ^]^ Although there are no one‐to‐one chemical bonds formation, the vdW interaction between the overlapped edges of graphene and TMDC materials could also guide the TMDC orientations under well‐controlled conditions.^[^ ^174^, ^175^ ^]^

## 4 Conclusion and Outlook

**Figure 10:**
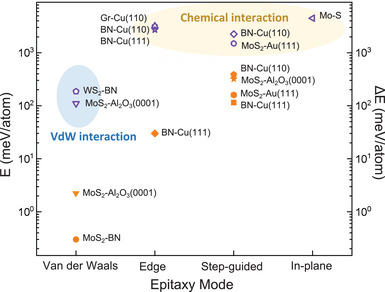
Binding energy *E* (left axis) between 2D domains with most preferred orientation and the substrate (purple) and the binding energy differences Δ*E* (right axis) for 0^○^ and 60^○^ oriented domains (orange) in different epitaxy modes.

After more than 10 years’ intense study, the CVD growth of single‐crystal 2D materials has achieved great improvement, from the micrometer‐scale single domain to wafer‐scale single‐crystal film. Although the single nucleus method can also obtain inch‐size single crystal, the epitaxial growth of 2D single crystals on single‐crystal substrates is more simple, economical and practical to realize the large‐scale manufacturing. The four epitaxial growth modes discussed in this review, vdW epitaxy, edge epitaxy, step‐guided epitaxy and in‐plane epitaxy, feature distinct interactions between 2D materials and substrates and the epitaxy mechanisms (**Figure** **10**). In vdW epitaxy, the interaction is weak vdW or quasi vdW force, and the epitaxy follows rotation‐alignment behavior where the nuclei with random orientations could rotate to the aligned ones driven by binding energy difference between different rotation angles. But the energy difference is small due to the weak vdW interaction, thus the growth window for vdW epitaxy is narrow in practice. Generally, the epitaxial growth of vertical 2D heterojunctions or 2D materials on nonmetal substrates follows vdW epitaxy. The commonly used transitional metal substrates could interact with the edge of the 2D materials due to their catalytic activities, thus resulting in the edge epitaxy or step‐guided epitaxy. In edge epitaxy, the epitaxial relation between the 2D material and substrate is the same with that in vdW epitaxy, while due to the strong chemical bonding between 2D edge and metal, the 2D lattice orientation is determined when nucleation begins. And this is also true for step‐guided epitaxy. The strong interactions between the 2D domains and metal substrates in the case of edge epitaxy and step‐guided epitaxy also lead to much lower formation energy of the nuclei with preferred orientation, making the edge and step‐guided epitaxy are more robust. For 2D materials with lower symmetry like h‐BN and TMDCs, there always exist two preferred orientations (0° and 60°) due to their small energy differences in the vdW and edge epitaxy, while the steps on the substrate surface could lift off the energy difference and induce the unidirectional alignment in step‐guided epitaxy. Furthermore, the energy difference is much larger in the case of high‐index metal substrates with steps trending only down on the entire surface than that in the case of low‐index metal substrates with steps trending both up and down. That means the unidirectional alignment is more feasible on the high‐index metal substrate. In addition, sapphire substrates can also be applied to achieve the step‐guided epitaxial growth of TMDC materials. But the high stability of sapphire at growth temperature makes the stitching of TMDC domains more difficult than that on the metal substrates, requiring very careful control of the growth kinetics. In‐plane epitaxy is actually more like conventional one, requiring restrict lattice match, but with 1D coherent interface. In general, the experimental window for achieving in‐plane epitaxial growth is large due to the strong chemical bonding at the epitaxial interface. By the delicate design and careful control, in‐plane epitaxy can even realize in‐plane multi‐heterostructure and superlattice. In fact, in‐plane epitaxy is the only technique to prepare in‐plane heterojunctions.

Up to now, meter‐scale graphene and h‐BN and wafer‐scale TMDC monolayer single crystals have been successfully prepared on single‐crystal metal or sapphire substrates by the CVD method. The large‐scale fabrication and applications of 2D single crystals seem to be at the corner. But the growth behaviors especially the nucleation of 2D materials has not been understood thoroughly, which makes the precise and controllable growth difficult. Besides, the growth of multilayer or the vertical heterojunction of 2D materials is still faced with many problems, like the layer number control, stacking angle control and lateral continuity control. The prerequisite of controllable growth of multilayer or the vertical heterojunction is the understanding of growth mechanism, but it is still very limited at the present because of the more complex processes. At the early days, most of understanding to the growth mechanism was gained by inferring from the ex‐situ experimental results. But we know the best way to figure out what happens is to “see” what is happening. Thus, the in situ growth and characterization technologies have attracted more and more attentions nowadays. MOCVD and molecule beam evaporation (MBE), which could realize more accurate control over the growth process and have been demonstrated to have potential to prepare high‐quality 2D single‐crystal films,^[^ ^110^, ^176^ ^]^ always possess in situ characterization capability and thus are very important techniques to explore the growth mechanism in the future. Besides, thanks to the quick technology advances, currently some in situ technologies, such as in situ TEM and STM, can achieve atomic‐scale spatial resolution and femtosecond‐scale time resolution.^[^ ^177^, ^178^ ^]^ We believe by combining in situ growth and characterization technologies with theoretical calculations, deeper insight in 2D materials growth would be achieved in the near future, and precise and controllable growth of monolayer, multilayer and heterojunctions of 2D single crystals would be realized. This will greatly promote the high‐end industrial application of 2D materials and also provide new opportunities for the growth of many other materials on 2D single crystals.

## Conflict of Interest

The authors declare no conflict of interest.
